# A Validated Trigonelline-Based Method for the Standardization and Quality Control of *Trigonella foenum-graecum* L.

**DOI:** 10.12688/f1000research.157659.2

**Published:** 2024-12-04

**Authors:** Mohammed Aldholmi, Rizwan Ahmad, Salma Hago, Ali Alabduallah

**Affiliations:** 1 Department of Natural Products, College of Clinical Pharmacy, Imam Abdulrahman Bin Faisal University, Dammam, Eastern Province, Saudi Arabia; 2 Faculty of Pharmacy, Department of Pharmacognosy, University of Gezira, Wad Madani, Gezira, Sudan

**Keywords:** Fenugreek, UPLCMSMS, green, extraction, analysis

## Abstract

**Background:**

Fenugreek, or *Trigonella foenum-graecum* L, is an edible and medicinal plant of the Fabaceae family. Fenugreek seeds are rich in proteins, lipids, and essential nutrients, and they also contain various phytochemicals, including flavonoids, steroidal saponins, coumarin, and alkaloids such as trigonelline. Trigonelline (TG) is a bioactive plant alkaloid initially extracted from fenugreek seeds. A substantial portion of fenugreek’s health benefits may rely on the presence of TG. This study addresses the critical need for a fast, green, and economical method that overcomes inefficiencies, high solvent usage, and sensitivity limitations in the quantification of TG.

**Methods:**

Fenugreek seeds from various origins were extracted using three green solvents: acetone (ACt), ethanol (EtOH), and water (H _2_O). The UPLC-MS/MS method was developed and validated using a green mobile phase of H _2_O: EtOH, and an r ^2^-value of 0.999 in the linearity range of 0.1-500 ppb was adopted. The method was validated with an accuracy of 98.6% for trace analysis of TG using a small amount (10 mg) of fenugreek samples from five different origins.

**Results:**

The average extract yield was 5.36 mg/100 mg with a standard deviation (SD) of 6.3, with the highest extract yield observed in H _2_O. The ESI (+ve) of the UPLC-MS/MS resulted in the fragmentation pattern ( *m/z*) 138→94.10→92.05→78.20. The TG quantification revealed an average TG concentration of 181.4 ppb (SD = 176.4), with the highest amount of TG in H _2_O extract (mean = 392.7, SD = 132.4 ppb), followed by EtOH (mean = 91.9, SD = 83.3 ppb) and ACt (mean = 59.5, SD = 30.9 ppb). The TG amount observed in the validation step substantiated the efficiency and reproducibility of the developed method.

**Conclusions:**

The method may be used as an effective tool for a green, rapid, economical, and eco-friendly extraction and quantification of TG in diverse matrices of pharmaceutical, cosmeceutical, herbal, and food products.

## Introduction

*Trigonella foenum-graecum* L. (fenugreek) is a medicinal and food plant that belongs to the family *Fabaceae*, which includes common medicinal and food plants such as *Pisum sativum* (pea), *Cicer arietinum* (chickpeas), *Phaseolus* (beans), *Glycine max* (soybean), *Arachis hypogaea* (peanut), and *Glycyrrhiza glabra* (licorice). ^1^ Fenugreek is cultivated in several countries worldwide, including the Mediterranean region, North Africa, and India. ^2^ The seed is consumed as a condiment and seasoning in food preparations and is believed to possess nutritional and health benefits. ^2^ For several centuries, fenugreek has been consumed orally as a traditional treatment for a wide range of diseases, including diabetes, fever, and abdominal colic, and applied topically for abscesses, boils, and carbuncles. ^3^ Various pharmacological and clinical studies have confirmed significant medicinal properties of fenugreek seeds, including antidiabetic activity, anti-obesity activity, hypolipidemic activity, anticancer activity, antioxidant activity, anti-inflammatory activity, and antibacterial activity due to the presence of numerous bioactive components. ^4^ Fenugreek seeds contain several classes of compounds, including proteins, lipids, amino acids, vitamins, minerals, galactomannan fibre (a carbohydrate), flavonoids, steroidal saponins, coumarin, and alkaloids such as trigonelline (TG). ^2^

**Figure 1.**
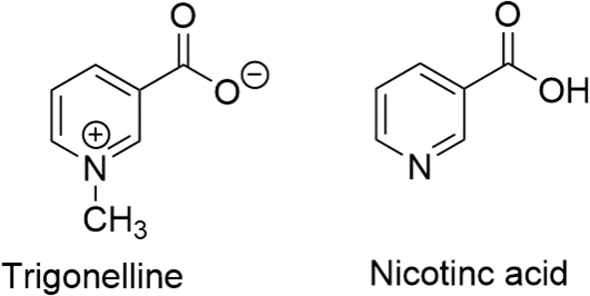
Chemical structures of trigonelline and nicotinic acid.

TG is a bioactive plant alkaloid that was first isolated from fenugreek seeds but is also abundant in other plant-derived food products, such as coffee beans. ^5^ It is an N-methylated form of nicotinic acid or vitamin B _3_ ( Figure 1), hence chemically known as N-methylnicotinic acid (C _7_H _7_NO _2_). This fenugreek alkaloid has been reported to have antidiabetic, antihyperlipidemic, antimigraine, sedative, antibacterial, antiviral, anticancer, and phytoestrogenic effects; also it inhibits platelet aggregation and improves memory function. ^2, 4^ It has been reported to demonstrate its antidiabetic efficacy by enhancing the insulin signalling pathway, alleviating endoplasmic reticulum stress, and reducing oxidative damage in individuals with type 2 diabetes. ^4^ TG has been shown to enhance the axonal formation of neurons and recover memory function in Alzheimer’s disease model mice. ^6^ It has also been demonstrated to incorporate into the nicotinamide adenine dinucleotide (NAD+) pool and increase NAD+ levels, improving muscle function during ageing. ^5^ Therefore, this fenugreek bioactive compound appears to be a significant contributor to the medicinal properties of fenugreek seeds.

Since a significant part of the fenugreek health benefits potentially depends on the presence of TG in the utilised product, there is a critical need to develop TG-based extraction and analysis methods for the standardization and quality control of fenugreek products to ensure their efficacy and safety. The optimal extraction and analysis methods should be fast, efficient, and accurate, using an economical, eco-, and human-friendly approach. Additionally, the developed methods should avoid the use of high temperatures, as it has been reported that trigonelline breaks down to niacin at high temperatures. ^2^ Previous studies reported the use of HPLC-UV-Vis and LC-MS techniques for TG quantification, but they have the disadvantage of involving non-green and toxic solvents in the mobile phase system. ^7– 12^ Therefore, a loophole still exists for the extraction and determination of TG in fenugreek samples using a fast, green, economical, eco-friendly, and less toxic methodology. The current study aims to investigate the efficiency of three green solvents for extraction of TG from fenugreek using an ultrasonic dismembrator (20 kHz), develop an economical, eco-, and human-friendly analysis method, and practically apply the method to fenugreek samples from different origins.

## Methods

### Solvents, chemicals, and instruments

Analytical-grade acetone (ACt, Cat. 107021) and ethanol (EtOH, Cat. 107017), as well as HPLC-grade ethanol (EtOH, Cat. 111727), were purchased from Merck (Darmstadt, Germany). Water (H _2_O) used for extraction and LCMSMS analysis was produced in-house using Millipore equipment (Millipore, Bedford, MA, USA). The standard chemical trigonelline (TG, Cat. 1686411) was obtained from Sigma Aldrich St Louis, MO, USA.

Fisher Scientific (2000 Park Lane Pittsburgh, PA, USA) ultrasonics dismembrator [power (50 Watt), frequency (20 kHz), transducer (Model CL-334), horn (220 A), titanium probe (420 A; 1 mm diameter)] was used for extraction. Ultra-pressure liquid chromatography-mass spectrometry (UPLC-MS/MS) instrument (Shimadzu, Japan, LCMS-8050) consisting of binary pumps (LC-30AD), thermostatted column compartment (CTO30A), UV-DAD detector (SPD-M20A), and triple-quadrupole mass detector with electrospray-ionisation-source (TQMS-ESI) was used for quantitative analysis. The data was processed and analysed with the help of LabSolutions software (Kyoto, Japan V 5.93). The evaporation and drying of the solvents from the samples were accomplished with the help of a miVac Quattro Concentration System (Genevac Ltd., Ipswich, United Kingdom). The analytical column used for the separation was a C18 Acquity UPLC column from Waters (2.1 × 100 mm; 1.7 mm).

### Fenugreek samples collection and preparation

Dried fenugreek seed samples originating from five different countries consisting of India, Egypt, Iran, Saudi Arabia, and Yemen were collected from local markets in the Kingdom of Saudi Arabia, as reported in our previous study. ^13^ Three different types of fenugreek seeds (based on color and size) were collected for each origin, resulting in fifteen fenugreek samples (3 × 5 = 15).

### Green Ultrasonics Extraction (USE) for TG

Fenugreek seed samples were extracted for the three accessions from each origin using three green solvents of ACt, EtOH, and H _2_O, resulting in forty-five fenugreek extracts (3 × 5 × 3 = 45). The samples were properly coded from F1 to F15 plus A, B, and C letters standing for ACt, EtOH, and H _2_O, respectively. The extraction procedure was adopted with slight modification from the previously developed in-house USE method. ^14^ Briefly, 100 mg of the fenugreek seeds from each accession were weighed, and 10 mL of the respective solvent was added. The amplitude and pulse were set at 30% and 30/10 s, respectively. The five-minute extraction was followed by the evaporation of solvents using the miVac Quattro Concentration System (Genevac Ltd., Ipswich, United Kingdom). The dried samples were weighed for extract yield calculation (mg/100 mg).

### Green UPLC-MS/MS analytical method for TG

**Method development for green UPLC-MS/MS analysis**

The standard drug TG was dissolved (1 mg/mL) in HPLC-grade EtOH to prepare a stock solution. For working standards, the stock solution was further diluted in the required solvent volume to construct six points in the linearity range of 0.1-500 ppb (0.1, 5, 10, 50, 100, and 500 ppb). The extracts, stock, and working standard solutions were prepared in HPLC-grade EtOH and filtered through a syringe filter (0.2 mm). For separation and quantitation of TG, the mobile phase of H _2_O (A) and EtOH (B) was used to run the TG standard solutions with a gradient of 5-95% B using the C18 column. Mobile phase at different gradient and isocratic elution compositions, flow rates (0.1, 0.2, 0.3 mL/min), and injection volumes (1, 2, 5 mL) were tested for the efficient separation of TG.

The conditions used for mass spectrometry were optimised to obtain the most appropriate ionisation and fragmentation pattern. The ESI (electrospray ionisation) interface was used for the mass analysis, and a positive MRM (multiple reaction monitoring) mode was applied to quantify and analyse the daughter fragments using an external standard calibration curve. Following the successful fragmentation via optimal collision energy (CE) selection, the fragment (m/z) with the highest intensity (base peak), along with the retention time (Rt) of the peak, were used to identify the target analyte in all samples. The final LC and MS conditions are presented in Section Chromatographic separation and MS optimisation.

**Method validation for green UPLC-MS/MS analysis**

The UPLCMSMS-MD was validated in terms of accuracy, linearity, LOD (limit of detection), and LOQ (limit of quantification). The linearity of the method was evaluated using six different calibration points in the range of 0.1-500 ppb, where the regression equation and r ^2^-value were calculated. The accuracy, LOD, and LOQ were calculated via in-built LabSolutions software using the ICH guidelines ^15^ and previously validated parameters for the UPLC-MS/MS analytical method. ^16^

### Applicability of the USE-UPLC-MS/MS for trace analysis

The developed USE-UPLC-MS/MS was further tested in terms of efficiency and applicability of the method for trace analysis by H _2_O extraction and analysis of lower amounts (10 mg) of the fenugreek samples containing the lowest TG quantity from each origin (F1, F5, F8, F10, and F15), as mentioned previously. The extracted samples were dried, diluted, and prepared for UPLC-MS/MS analysis to determine TG concentrations.

## Results

### Extracts yield

**Determination of the extract yield in different fenugreek samples**

The extract yield for the forty-five fenugreek extracts ( *N=45*) exhibited a sum of 241.0 mg/4500 mg, with a mean of 5.36 (SD = 6.3). The range for the extract yield observed was (minimum to maximum) 1.1-30.6 mg ( *N=45*). For the average extract yield/origin ( *N=9:* mg/100 mg) *,* the descending order observed was Egypt (mean = 7.1, SD = 8.5 mg)> Yemen (mean = 6.6. SD = 9.2 mg)> Saudi Arabia (mean = 6.0, SD = 6.0 mg)> Iran (mean = 3.7, SD = 2.9 mg)> India (mean = 3.4, SD = 2.0 mg). With regard to the highest extract yield in individual fenugreek samples of each origin ( *N=9*), the following samples exhibited the highest extract yield in each origin: F3C in H _2_O (6.3 mg) for the Indian samples, F5C in H _2_O (21.5 mg) for the Saudi Arabian, F7A in ACt (9.6 mg) for the Iranian, F11C in H _2_O (28.1 mg) for the Egyptian, and F15C in H _2_O (30.6 mg) for the Yemeni origin. The fenugreek samples noted with the highest yield among the 45 samples were F15C (30.6 mg)> F11C (28.1 mg)> F5C (21.5 mg)> F11A (10.8 mg). The results of average and individual yields in different origins are summarized in Table 1.

**Table 1.** Average and individual extract yields for fenugreek samples (F1-F15) extracted with ACt (A), EtOH (B), and H _2_O (C).

Country of origin	Sample code	Extract yield (mg/100 mg)	Average Yield/origin (mg/100 mg)	SD
India	F1A	5.6	3.4	2.0
	F1B	1.3		
	F1C	1.1		
	F2A	5.3		
	F2B	2.1		
	F2C	4.2		
	F3A	3.0		
	F3B	1.8		
	F3C	6.3		
Saudi Arabia	F4A	4.3	6.0	6.0
	F4B	3.7		
	F4C	6.0		
	F5A	3.4		
	F5B	1.8		
	F5C	21.5		
	F6A	5.9		
	F6B	2.2		
	F6C	5.5		
Iran	F7A	9.6	3.7	2.9
	F7B	1.4		
	F7C	2.9		
	F8A	7.5		
	F8B	1.7		
	F8C	3.8		
	F9A	3.1		
	F9B	1.1		
	F9C	2.1		
Egypt	F10A	7.6	7.1	8.5
	F10B	1.4		
	F10C	4.9		
	F11A	10.8		
	F11B	4.2		
	F11C	28.1		
	F12A	3.1		
	F12B	1.1		
	F12C	2.4		
Yemen	F13A	3.2	6.6	9.2
	F13B	1.2		
	F13C	3.9		
	F14A	3.6		
	F14B	1.4		
	F14C	4.2		
	F15A	7.5		
	F15B	3.6		
	F15C	30.6		

**Variation of extract yield in green solvents**

For the green solvents, the sum and mean of extract yield in each solvent ( *N=15*) were 83.5 and 5.6 mg for ACt, 30.0 mg and 2.0 mg for EtOH, and 127.5 and 8.5 for H _2_O, respectively. The average extract yield for each individual origin in the extraction solvents resulted in the following descending order: India, mean = 4.6, SD = 1.4 mg (ACt) > mean = 3.9, SD = 2.6 mg (H _2_O) > mean = 1.7, SD = 0.4 mg (EtOH); Saudi Arabia, mean = 11.0, SD = 9.1 (H _2_O)> mean = 4.5, SD = 1.3mg (ACt)> mean = 2.6, SD = 1.0 mg (EtOH); Iran, mean = 6.7, SD = 3.3 mg (ACt)> mean = 2.9, SD = 0.9 mg (H _2_O)> mean = 1.4, SD = 0.3 mg (EtOH); Egypt, mean =11.8, SD = 14.2 mg (H _2_O)> mean = 7.2, SD = 3.9 mg (ACt)> mean 2.2, SD = 1.7 mg (EtOH)); and Yemen, mean = 12.9, SD = 15.3 mg (H _2_O)> mean = 4.8, SD = 2.4 mg (ACt)> mean = 2.1, SD = 1.3 mg (EtOH).

On an individual basis, the highest extract yield was observed for H _2_O (30.6 mg/100 mg) in the Yemeni fenugreek sample, followed by ACt (10.8 mg/100 mg) in the Egyptian fenugreek sample, and EtOH (3.7 mg/100 mg) in the Saudi Arabian fenugreek sample. The total and average extract yield data for the fenugreek samples suggest a descending order of extract yield with the superior solvent to be H _2_O>ACt> EtOH. The data for the extract yield in green solvents is shown in detail in Table 2.

**Table 2.** Yield/Origin (mg) and total extract yield (mg) in green solvents for fenugreek samples with SD.

Origin	Code	Yield in Act (A)			Yield in EtOH (B)			Yield in H _2_O (C)		
		Yield/sample	Average/Origin	SD	Yield/sample	Average/Origin	SD	Yield/sample	Average/Origin	SD
** India**	F1	5.6	4.6	1.4	1.3	1.7	0.4	1.1	3.9	2.6
	F2	5.3			2.1			4.2		
	F3	3.0			1.8			6.3		
**Saudi Arabia**	F4	4.3	4.5	1.3	3.7	2.6	1.0	6.0	11.0	9.1
	F5	3.4			1.8			21.5		
	F6	5.9			2.2			5.5		
** Iran**	F7	9.6	6.7	3.3	1.4	1.4	0.3	2.9	2.9	0.9
	F8	7.5			1.7			3.8		
	F9	3.1			1.1			2.1		
**Egypt**	F10	7.6	7.2	3.9	1.4	2.2	1.7	4.9	11.8	14.2
	F11	10.8			4.2			28.1		
	F12	3.1			1.1			2.4		
**Yemen**	F13	3.2	4.8	2.4	1.2	2.1	1.3	3.9	12.9	15.3
	F14	3.6			1.4			4.2		
	F15	7.5			3.6			30.6		
**Sum**	83.5				30.0			127.5		
**Mean**	5.6				2.0			8.5		
**SD**	2.5				1.0			9.7		

### Chromatographic separation and MS optimisation

**Figure 2.**
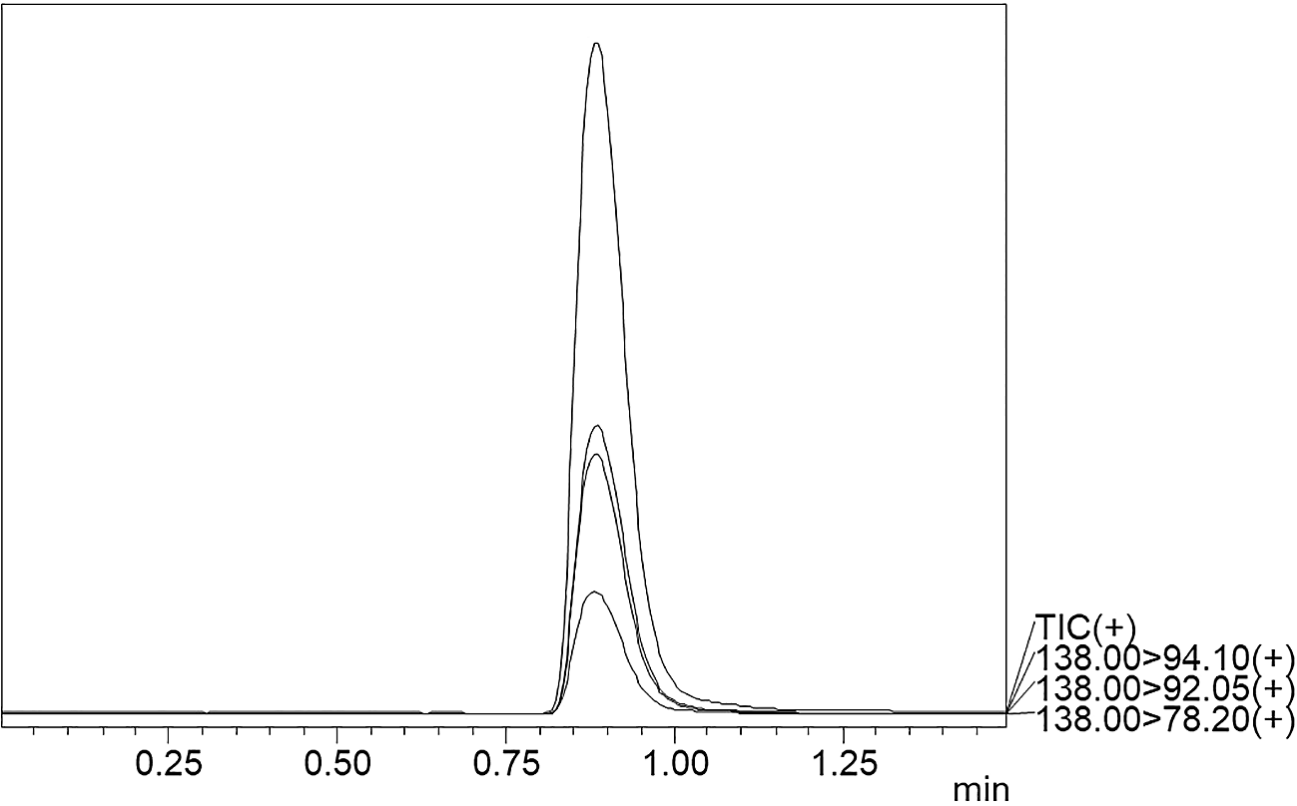
UPLC-MS/MS chromatogram of TG standard.

**Figure 3.**
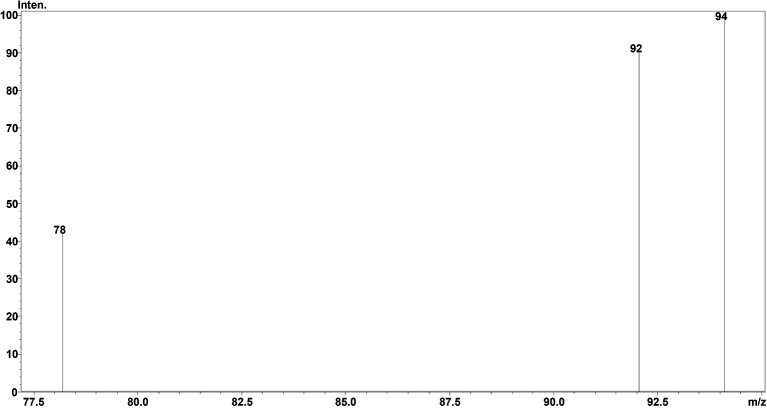
MSMS fragmentation pattern of TG ( *m/z*).

**Figure 4.**
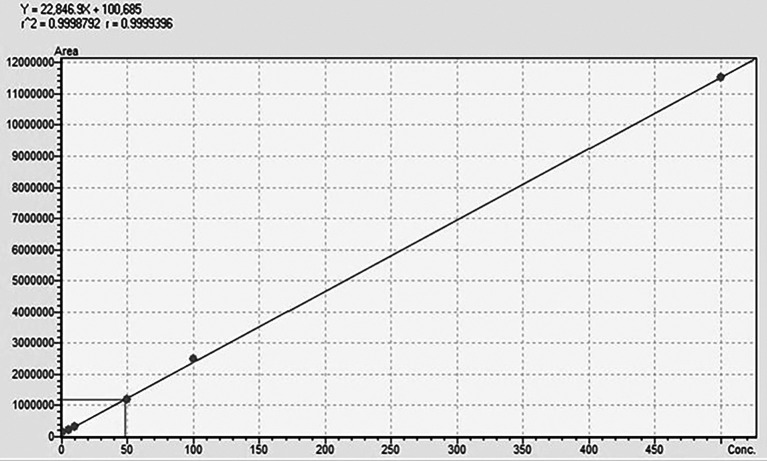
Standard calibration curve of TG.

The chromatographic method development resulted in a mobile phase of H _2_O (A)+EtOH (B) with an isocratic elution of 30 (A):70 (B). The retention time for TG was 0.882 min with a runtime of 1.5 min at a flow rate of 0.3 mL/min. The method was validated using ICH guidelines in the linearity range of 0.1-500 ppb, where an accuracy of 98.6% with an r ^2^-value of 0.999 was obtained, showing the efficiency and reproducibility of the method. For MS optimisation, a positive (+ve) MRM mode with an ESI interface resulted in the daughter fragments ( *m/z*) of 138 →94.10 →92.05 →78.20. The base peak of 94.10 was selected for the data analysis and calibration curve development using the six different concentrations of calibration points. The details regarding LC separation and MS optimisation are presented in Table 3. A representative chromatogram showing the Rt for the TG-peak is given in Figure 2 whereas the mass fragmentation pattern with the base peak for TG is shown in Figure 3. The details regarding the calibration curve, regression equation, and r ^2^-value with the linearity range points are presented in Figure 4.

**Table 3.** LC and MS conditions for the analysis of TG in fenugreek samples.

LC and MS parameters					
**Mobile phase**			H _2_O (A)+EtOH (B)		
**Mobile phase composition**			Isocratic at 30 (A):70 (B)		
**Flow rate (FR)**			0.3 mL/min		
**Rt (retention time)**			0.882 min		
**Runtime**			1.5 min		
**LOD (limits of detection)**			0.5 ppb		
**LOQ (limits of quantification)**			1.4 ppb		
**Accuracy**			98.6%		
**Regression equation**			Y=22846.9x+100685		
**r** ^**2**^ **-value**			0.999		
**Linearity range**			0.1-500 ppb		
**TG-fragmentation pattern**	**Precursor mass**	**Product m/z**	**Q1 pre-bias (V)**	**CE**	**Q3 pre-bias (V)**
	138	94.10	-12.0	-22.0	-17.0
	138	92.05	-10.0	-20.0	-22.0
	138	78.20	-13.0	-25.0	-14.0
**MRM mode**			+ve		
**Dwell time**			100 msec		
**Event time**			0.309		
**Mass end time**			1.5		
**Interface**			ESI		
**Nebulizing gas flow**			3 L/min		
**Drying gas flow**			10 L/min		
**Heating gas flow**			10 L/min		
**Interface temperature**			300°C		
**DL temperature**			250°C		
**Heat block temperature**			400°C		
**Interface current**			0.4 uA		

### TG quantification

**UPLC-MS/MS analysis of TG in different fenugreek samples**

The TG amount observed was within the range of (minimum to maximum) 22.2-535.0 ppb (N = 45). The sum, mean, and SD for the TG amount in the fenugreek samples ( *N* = 45) were 8161 ppb, 181.4, and 176.4 ppb, respectively. The average TG amount/individual origin ( *N=9;* ppb) created a descending order of Yemen (mean = 220.0, SD = 191.7)> India (mean = 211.3, SD = 198.1)> Saudi Arabia (mean = 190.8, SD = 210.7)> Egypt (mean = 189.5, SD = 165.2)> Iran (mean = 95.2, SD = 110.4). The greatest TG amounts were observed in the following fenugreek samples: 535.0 ppb for F3C (Indian)> 510.0 ppb for F6C (Saudi Arabian)> 491.0 ppb for F4C (Saudi Arabian)> 475.0 ppb for F14C (Yemeni).

For the highest TG amount in each individual origin (N = 9), an amount of 535.0 ppb in H _2_O was observed for F3C (India), 510.0 ppb in H _2_O for F6C (Saudi Arabia), 370.0 ppb in H _2_O for F9C (Iran), 423.0 ppb in H _2_O for F12C (Egypt), and 475.0 ppb in H _2_O for F14C (Yemen). Detailed data regarding the TG amount in 45 different samples of fenugreek seeds is provided in Table 4.

**Table 4.** Average and individual TG amounts in fenugreek samples (F1-F15) extracted with ACt (A), EtOH (B) and H _2_O (C).

Country of origin	Sample code	TG amount (ppb)	TG %	Average TG amount/origin (ppb)	SD
** India**	F1A	56.6	0.00011	211.3	198.1
	F1B	105.5	0.00021		
	F1C	434.0	0.00087		
	F2A	43.2	0.00009		
	F2B	146.0	0.00029		
	F2C	437.0	0.00087		
	F3A	36.7	0.00007		
	F3B	108.1	0.00022		
	F3C	535.0	0.00107		
**Saudi Arabia**	F4A	37.0	0.00007	190.8	210.7
	F4B	46.7	0.00009		
	F4C	491.0	0.00098		
	F5A	80.4	0.00016		
	F5B	62.3	0.00012		
	F5C	405.0	0.00081		
	F6A	34.3	0.00007		
	F6B	50.1	0.00010		
	F6C	510.0	0.00102		
** Iran**	F7A	27.3	0.00005	95.2	110.4
	F7B	22.2	0.00004		
	F7C	92.6	0.00019		
	F8A	22.6	0.00005		
	F8B	24.3	0.00005		
	F8C	82.9	0.00017		
	F9A	133.2	0.00027		
	F9B	81.7	0.00016		
	F9C	370.0	0.00074		
**Egypt**	F10A	100.7	0.00020	189.5	165.2
	F10B	107.5	0.00022		
	F10C	391.0	0.00078		
	F11A	47.2	0.00009		
	F11B	35.4	0.00007		
	F11C	407.0	0.00081		
	F12A	91.6	0.00018		
	F12B	102.3	0.00020		
	F12C	423.0	0.00085		
**Yemen**	F13A	49.1	0.00010	220.0	191.7
	F13B	102.7	0.00021		
	F13C	452.0	0.00090		
	F14A	74.3	0.00015		
	F14B	359.2	0.00072		
	F14C	475.0	0.00095		
	F15A	58.4	0.00012		
	F15B	24.4	0.00005		
	F15C	385.0	0.00077		

**Variation of TG amount in green solvents**

The UPLC-MS/MS analysis of the green extracts of fenugreek seeds exhibited the highest average amount of TG ( *N=15*) in H _2_O (mean = 392.7, SD = 132.4 ppb) followed by EtOH (mean = 91.9, SD = 83.3 ppb) and ACt (mean = 59.5, SD = 30.9 ppb). On an individual basis, the highest amount of TG was detected in H _2_O extracts of all fenugreek samples ( *N = 45*) when compared to the EtOH and ACt extracts of the same samples.

For the average TG amount in the three solvent extracts of each individual origin, a descending order may be constructed ( *N=9*) as follows *:* India, mean = 468.7, SD = 57.5 ppb (H _2_O)> mean = 119.9, SD = 22.7 ppb (EtOH)> mean = 45.5, SD = 10.1 ppb (ACt); Saudi Arabia, mean 468.7, SD = 55.9 ppb (H _2_O)> mean = 53.0, SD = 8.2 ppb (EtOH)> mean = 50.6, SD = 25.9 ppb (ACt); Iran, mean = 181.8, SD = 163.0 ppb (H _2_O)> mean = 61.0, SD = 62.6 ppb (ACt)> mean = 42.7, SD = 33.8 ppb (EtOH); Egypt, mean = 407.0, SD = 16.0 ppb (H _2_O)> mean = 81.7, SD = 40.2 ppb (EtOH)> mean = 9.8, SD = 28.6 ppb (ACt); and Yemen, mean = 437.3, SD = 46.8 ppb (H _2_O)> mean = 162.1, SD = 175.1 ppb (EtOH)> mean = 60.6, SD = 12.8 ppb (ACt). The average and individual TG amounts suggest the descending order for solvents with optimal TG amount to be H _2_O> EtOH> ACt. The data regarding the TG amount is provided in detail in Table 5.

**Table 5.** TG amount/Origin (ppb) and total TG amount (ppb) quantified via UPLC-MS/MS in the green extracts of fenugreek.

Origin	Code	TG in Act (A)			TG in EtOH (B)			TG in H _2_O (C)		
		TG/sample	Average/Origin	SD	TG/sample	Average/Origin	SD	TG/sample	Average/Origin	SD
India	F1	56.6	45.5	10.1	105.5	119.9	22.7	434.0	468.7	57.5
	F2	43.2			146.0			437.0		
	F3	36.7			108.1			535.0		
Saudi Arabia	F4	37.0	50.6	25.9	46.7	53.0	8.2	491.0	468.7	55.9
	F5	80.4			62.3			405.0		
	F6	34.3			50.1			510.0		
Iran	F7	27.3	61.0	62.6	22.2	42.7	33.8	92.6	181.8	163.0
	F8	22.6			24.3			82.9		
	F9	133.2			81.7			370.0		
Egypt	F10	100.7	79.8	28.6	107.5	81.7	40.2	391.0	407.0	16.0
	F11	47.2			35.4			407.0		
	F12	91.6			102.3			423.0		
Yemen	F13	49.1	60.6	12.8	102.7	162.1	175.1	452.0	437.3	46.8
	F14	74.3			359.2			475.0		
	F15	58.4			24.4			385.0		
Sum	892.6				1378.4			5890.5		
Mean	59.5				91.9			392.7		
SD	30.9				83.3			132.4		

### Applicability of USE-UPLC-MS/MS for trace analysis

**Figure 5.**
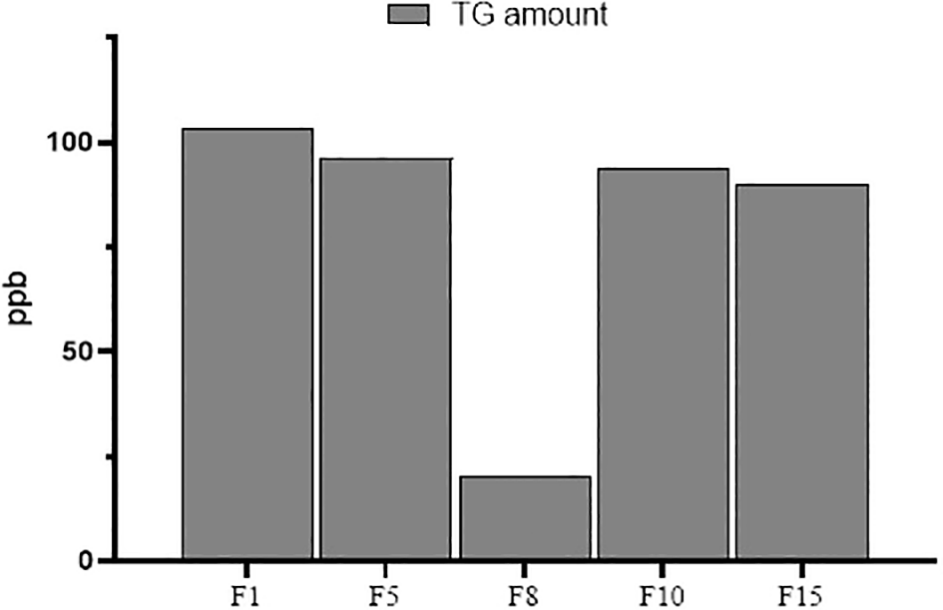
Trace analysis of TG in 10 mg of five fenugreek samples.

The developed USE-UPLC-MS/MS was further tested in terms of the efficiency and applicability of the method for trace analysis by H _2_O extraction and analysis of lower amounts (10 mg) of fenugreek samples. A sample from each origin (F1 for India, F5 for Saudi Arabia, F8 for Iran, F10 for Egypt, and F15 for Yemen) was selected for this purpose. The developed USE-UPLC-MS/MS was able to extract and detect TG in the low-amount samples (10 mg) with a minimum amount of 20 ppb in F8 (Iran). The TG amount from the 10 mg extracts of fenugreek samples ( *N=5*) showed a sum of 403.4 ppb with a mean of 80.68 (SD = 34.28 ppb). The TG amount ranged from a minimum of 20 ppb to a maximum amount of 103.5 ppb. The data for the TG amounts in all five samples is presented in Figure 5.

## Discussion

The USE protocol was selected for extraction due to its numerous advantages, including high extraction efficiency and the use of the least possible sample amount, solvent volume, and extraction time. ^14, 16^ The USE method used three green solvents (ACt, EtOH, and H _2_O) to assess the optimal solvent with the highest TG amount. Compared to toxic solvents, these green solvents significantly reduce health risks, environmental contamination, and regulatory challenges, while also addressing consumer demands for sustainable and safe extraction methods. The combined benefits of these solvents highlight their superiority as extraction solvents, particularly for food bioactive compounds like trigonelline in fenugreek. The extraction results, represented by the individual and total extract yield, suggested H _2_O as an appropriate solvent for extraction. This study reports a high aqueous extract yield and TG amount for H _2_O among the three green solvents, which agrees with previous studies reporting H _2_O as an efficient extraction medium for TG. ^7, 8^ The total extract yield for the green solvents exhibited an order of H _2_O>ACt>EtOH. Moreover, the highest yield on an individual basis was observed for H _2_O in the Yemeni fenugreek sample. The aim of this study was to quantify the TG amount in these fenugreek samples; hence, an advanced analytical instrument of UPLC-MS/MS was employed to develop and validate a green in-house method for TG trace quantification.

A rapid, sensitive, green, and reproducible method was developed using a green mobile phase of H _2_O and EtOH with a low flow rate of 0.3 mL/min in a short runtime of 1.5 min with equilibration. The developed method was validated for accuracy, detection and quantification limits, and coefficient value determination as per ICH guidelines, where all the results were obtained within the specified limits. To the best of our knowledge, this is the first green, sensitive, and rapid UPLC-MSMS analysis method for the quantification of TG in fenugreek samples. Previous studies reported the use of HPLC-UV-Vis and LC-MS techniques for TG quantification in coffee beans, fenugreek, and dietary supplements available in the market, but non-green and toxic solvents were used in the mobile phase system. ^7– 10^ Therefore, the main strength of this study is the use of green solvents for extraction and analysis of TG in fenugreek samples. A successful green and rapid extraction and analysis method was developed in this study.

Following a successful UPLC-MS/MS, the extracted samples were analysed using the in-house method. A similar pattern was observed for the extracted samples, where the highest TG amounts were observed in the H _2_O samples. A descending order for the solvent with the highest quantities was constructed for all the forty-five extracted samples as H _2_O> EtOH> ACt. Previous studies have reported trigonelline to be freely soluble in water. ^7– 12^ Therefore, the solubility and extraction behaviour for TG in the aqueous medium complements the concept of TG aqueous solubility, which corroborates the outcomes.

This is the first time to report a comprehensive assessment of TG in fifteen samples from different origins marketed in Saudi Arabia using a green, rapid, efficient, and reliable method of USE-UPLC-MS/MS. The method assessed the quality of the fenugreek samples in terms of their TG concentration, where significant variation was observed among the samples from different origins. Previous TG quantification studies were conducted using coarsely powdered fenugreek samples, as demonstrated in a study by Bakhtiar et al. (2024) in Iran. ^17^ In that study, the fenugreek samples were analyzed using HPLC–PDA, and the average TG concentrations ranged between 0.426% and 0.678%. The variation in concentration was attributed to differences in geographical location and extraction procedures. In another study, the trigonelline content varied between 0.74% and 0.97%. ^18^ The high TG concentrations reported in previous studies might be explained by the preparation and crushing of fenugreek seeds into powder, which facilitates more efficient extraction.

The real challenge is to validate the efficiency and reproducibility of the developed USE-UPLC-MS/MS method to be applied practically for the assessment and evaluation of low concentrations of target analytes in complex matrices. At times, the challenge of trace analysis for TG in samples of dietary supplements or marketed products may be encountered; hence, it becomes essential to authenticate the scope of the developed method for quantification of TG at low amounts in a matrix. To prepare the method for the mentioned challenge, an extra validation step was undertaken for extraction and trace analysis of TG. A small amount (10x lower in concentration) of the fenugreek amount for the five different origin samples was weighed and extracted with USE as previously described. The extracted samples were diluted and analyzed via the UPLC-MS/MS analytical method. The quantification of TG was successfully accomplished at the lowest TG concentration present in the tested fenugreek samples, ensuring the applicability and reliability of the developed method.

The USE-UPLC-MS/MS method developed in this study extends the benefit of using TG as a marker for the quality control and standardization of any pharmaceutical, cosmeceutical, herbal, and dietary products containing TG. The method avoids the need for additional steps, such as seed crushing and sieving, by using an ultrasonic dismembrator. Furthermore, the method employs small sample amounts (10 mg) and low solvent volumes, ensuring cost-efficiency and eco-friendliness. Another add-on advantage is the use of H _2_O as a medium for extraction, which is usually produced within the labs using Millipore apparatus. Overall, this method offers high levels of convenience, reliability, sensitivity, and accuracy for the quality evaluation and standardization of any TG-containing samples.

## Conclusions

The study developed a green, fast, economical, and reliable method (USE-UHPLCMSMS) for the extraction and analysis of TG in different fenugreek seed extracts collected from different geographical origins. The method was further validated to ensure the efficiency in matrices containing trace amounts of TG. The use of water with the lowest possible amount of samples is considered a superior economical point of the developed extraction method, in addition to the green and short analysis time of the UPLC-MS/MS analytical method. The USE-UPLC-MS/MS method may serve as a tool in research labs and for the quality control and standardization of TG amounts in different commercial herbal products and dietary supplements.

## Ethics and consent

The present study did not involve the use of human or animals; therefore, ethical approval and consent were not required.

## Authors’ contributions

Conceptualization, S.H., M.A., and R.A.; Methodology, M.A., R.A. and S.H.; Software, M.A. and R.A.; Validation, M.A., S.H. and R.A.; Formal Analysis, M.A. and R.A.; Investigation, A.A. and R.A.; Resources, M.A. and R.A.; Data Curation, S.H. and R.A.; Writing—Original Draft Preparation, M.A., R.A., A.A. and S.H.; Writing—Review and Editing, M.A., R.A., S.H. and A.A.; Visualization, M.A., S.H. and R.A.; Supervision, S.H. and R.A.; Project Administration, S.H. and R.A. All authors have read and agreed to the final version of the manuscript.

## Data Availability

Zenodo: A validated trigonelline-based method for the standardization and quality control of Trigonella foenum-graecum L., https://doi.org/10.5281/zenodo.13991270. ^19^ This project contains the following underlying data:
• Raw data for extract yields and trigonelline amounts in fenugreek (extract yields and trigonelline amounts in 45 fenugreek samples). • Trigonelline LC-MSMS data (mass spectrum of trigonelline). Data are available under the terms of the Creative Commons Attribution 4.0 International license (CC-BY 4.0).

## Software availability statement

LabSolutions is proprietary software used for the analysis of mass spectra and the calculation of method validation parameters, such as the regression equation, coefficient of determination (R ^2^), accuracy, linearity, limit of detection (LOD), and limit of quantification (LOQ). The mass spectral data analysis and method validation calculations presented in this article, originally performed with LabSolutions, can alternatively be conducted using the freely accessible software LibreOffice version 24.8.2.

License: OSI approved open license software is under the Mozilla Public License (MPL) 2.0.
