# Supplementary material for: A Validated Trigonelline-Based Method for the Standardization and Quality Control of Trigonella foenum-graecum L
Source: F1000Res. 2024 Dec 18;13:1350. Originally published 2024 Nov 11. [Version 3] doi: 10.12688/f1000research.157659.3 (PMC11809625; doi:10.12688/f1000research.157659.3)
Supplement: Peer review history

# Reviewer report: Reviewer response for version 3

**DOI:** 10.5256/f1000research.175958.r350252

**Published:** 2024-12-31

**Authors:** Abdul Sadiq

**Affiliations:** 1 University of Malakand, Chakdara, Khyber Pakhtunkhwa, Pakistan

I approve this manuscript

Is the work clearly and accurately presented and does it cite the current literature?

Yes

If applicable, is the statistical analysis and its interpretation appropriate?

Yes

Are all the source data underlying the results available to ensure full reproducibility?

Yes

Is the study design appropriate and is the work technically sound?

Yes

Are the conclusions drawn adequately supported by the results?

Yes

Are sufficient details of methods and analysis provided to allow replication by others?

Yes

Reviewer Expertise:

Pharmaceutical Sciences

I confirm that I have read this submission and believe that I have an appropriate level of expertise to confirm that it is of an acceptable scientific standard.

# Reviewer report: Reviewer response for version 3

**DOI:** 10.5256/f1000research.175958.r350251

**Published:** 2024-12-23

**Authors:** Abdulrahman Koshak

**Affiliations:** 1 King Abdulaziz University, Jeddah, Saudi Arabia

I confirm that the authors have addressed and resolved all comments on the manuscript, and I approve it for indexing.

Is the work clearly and accurately presented and does it cite the current literature?

Yes

If applicable, is the statistical analysis and its interpretation appropriate?

Not applicable

Are all the source data underlying the results available to ensure full reproducibility?

Yes

Is the study design appropriate and is the work technically sound?

Yes

Are the conclusions drawn adequately supported by the results?

Yes

Are sufficient details of methods and analysis provided to allow replication by others?

Yes

Reviewer Expertise:

Pharmacognosy and natural products

I confirm that I have read this submission and believe that I have an appropriate level of expertise to confirm that it is of an acceptable scientific standard.

# Reviewer report: Reviewer response for version 2

**DOI:** 10.5256/f1000research.175377.r345766

**Published:** 2024-12-12

**Authors:** Abdulrahman Koshak

**Affiliations:** 1 King Abdulaziz University, Jeddah, Saudi Arabia

The manuscript is much clearer now; however, there are still two minor comments to address:

1. Under the section *Method Validation for Green UPLC-MS/MS Analysis*, what do you mean by "UPLCMSMS-MD"? Is it the same as UPLC-MS/MS? Please clarify.
2. It is evident that the percentages of TG differ significantly from those reported in the literature. You attributed this to the use of unprocessed seeds, which is a valuable observation. I suggest emphasizing the importance of seed crushing in releasing the active constituents of the plant. Additionally, you might want to highlight that certain traditional preparation methods, such as making Trigonella tea from uncrushed seeds, could result in a limited extraction of TG.

Is the work clearly and accurately presented and does it cite the current literature?

Yes

If applicable, is the statistical analysis and its interpretation appropriate?

Not applicable

Are all the source data underlying the results available to ensure full reproducibility?

Yes

Is the study design appropriate and is the work technically sound?

Yes

Are the conclusions drawn adequately supported by the results?

Yes

Are sufficient details of methods and analysis provided to allow replication by others?

Yes

Reviewer Expertise:

Pharmacognosy and natural products

I confirm that I have read this submission and believe that I have an appropriate level of expertise to confirm that it is of an acceptable scientific standard, however I have significant reservations, as outlined above.

# Response

**Published:** 2024-12-13

**Authors:** Salma Hago

**Affiliations:** Pharmacognosy, University of Gezira, Sudan

Response to the reviewer comment No. 1:

 This typo error and we modified it in the manuscript into UPLC-MS/MS

 Response to the reviewer comment No. 2

 The higher TG concentration observed in these previous studies can be explained by the preparation and crushing of the fenugreek sample into powder, which facilitates more efficient solvent penetration and extraction of bioactive compounds like TG. In contrast, the current study used intact seed to mimic traditional preparation methods where crushing and grinding may not be performed. This highlights the potential limitations of traditional methods such as making fenugreek tea, which result in lower TG extraction yield. Further, the findings emphasise the critical role of the seed preparation in maximising the release of active constituents and underscore the importance of considering these factors when comparing TG concentrations across different studies and extraction methods.

# Reviewer report: Reviewer response for version 2

**DOI:** 10.5256/f1000research.175377.r345767

**Published:** 2024-12-06

**Authors:** Abdul Sadiq

**Affiliations:** 1 University of Malakand, Chakdara, Khyber Pakhtunkhwa, Pakistan

I approve the manuscript in the current form

Is the work clearly and accurately presented and does it cite the current literature?

Yes

If applicable, is the statistical analysis and its interpretation appropriate?

Yes

Are all the source data underlying the results available to ensure full reproducibility?

Yes

Is the study design appropriate and is the work technically sound?

Yes

Are the conclusions drawn adequately supported by the results?

Yes

Are sufficient details of methods and analysis provided to allow replication by others?

Yes

Reviewer Expertise:

Pharmaceutical Sciences

I confirm that I have read this submission and believe that I have an appropriate level of expertise to confirm that it is of an acceptable scientific standard.

# Reviewer report: Reviewer response for version 1

**DOI:** 10.5256/f1000research.173145.r340136

**Published:** 2024-11-26

**Authors:** Abdul Sadiq

**Affiliations:** 1 University of Malakand, Chakdara, Khyber Pakhtunkhwa, Pakistan

The manuscript presents a scientifically sound and innovative method for the extraction and analysis of trigonelline in fenugreek seeds. The approach is environmentally sustainable, reproducible, and suitable for widespread application. Minor enhancements could be considered for future work, but the study meets the standards for publication. However, there are a few minor areas where clarity or wording could be slightly improved for better readability:

**Abstract:**

 "Fenugreek seeds contain a variety of phytochemicals, including proteins, lipids, amino acids, vitamins, flavonoids, steroidal saponins, coumarin, and alkaloids."

 Phytochemicals usually refer to biologically active compounds produced by plants, which usually include non-nutritive substances such as alkaloids, flavonoids, phenolic acids, tannins, terpenes, and saponins. If proteins or amino acids are being mentioned in the context of plant composition, it is more accurate to separate them from the term "phytochemicals." A clearer phrasing for the description of fenugreek seeds might be:

 "Fenugreek seeds are rich in proteins, lipids, and essential nutrients, and they also contain various phytochemicals, including flavonoids, steroidal saponins, coumarin, and alkaloids such as trigonelline."

**Introduction:**

 "Fenugreek is cultivated in several countries worldwide, including the Mediterranean region, North African areas, India, and other countries."

 The phrase "and other countries" is unnecessary after "including." Consider revising to avoid redundancy.

**Methodology:**

 "The USE protocol was selected due to the range of advantages USE presents, including high extraction efficiency and the use of the least possible sample amount, solvent volume, and extraction time."

 The phrase "the range of advantages USE presents" could be simplified to "the numerous advantages of USE."

**Conclusion:**

 "The method avoids the need for an extra step of seed crushing and sieving via the use of an ultrasonic dismembrator with small amounts of sample (10 mg) and low solvent volumes."

 Consider breaking this into two sentences for clarity, as the crushing and sieving highlight the simplicity of the method, while the use of small sample amounts and solvent volumes emphasizes cost-efficiency and eco-friendliness.

Is the work clearly and accurately presented and does it cite the current literature?

Yes

If applicable, is the statistical analysis and its interpretation appropriate?

Yes

Are all the source data underlying the results available to ensure full reproducibility?

Yes

Is the study design appropriate and is the work technically sound?

Yes

Are the conclusions drawn adequately supported by the results?

Yes

Are sufficient details of methods and analysis provided to allow replication by others?

Yes

Reviewer Expertise:

Pharmaceutical Sciences

I confirm that I have read this submission and believe that I have an appropriate level of expertise to confirm that it is of an acceptable scientific standard, however I have significant reservations, as outlined above.

# Response

**Published:** 2024-11-27

**Authors:** Salma Hago

**Affiliations:** Pharmacognosy, University of Gezira, Sudan

**Reviewer Comment 1:** Phytochemicals usually refer to biologically active compounds produced by plants, which typically include non-nutritive substances such as alkaloids, flavonoids, phenolic acids, tannins, terpenes, and saponins. If proteins or amino acids are mentioned in the context of plant composition, it is more accurate to separate them from the term "phytochemicals." A clearer phrasing for the description of fenugreek seeds might be: "Fenugreek seeds are rich in proteins, lipids, and essential nutrients, and they also contain various phytochemicals, including flavonoids, steroidal saponins, coumarin, and alkaloids such as trigonelline."  

**Response:** We agree with the reviewer’s comment regarding the definition of phytochemicals. The sentence in the abstract has been revised to separate proteins and amino acids from the phytochemical category for accuracy. The updated sentence now reads: "Fenugreek seeds are rich in proteins, lipids, and essential nutrients, and they also contain various phytochemicals, including flavonoids, steroidal saponins, coumarin, and alkaloids such as trigonelline."

**Reviewer Comment 2**: "Fenugreek is cultivated in several countries worldwide, including the Mediterranean region, North African areas, India, and other countries." The phrase "and other countries" is unnecessary after "including." Consider revising to avoid redundancy.  

**Response: **We appreciate the reviewer’s suggestion to avoid redundancy. The sentence has been revised to remove "and other countries" for conciseness. It now reads: "Fenugreek is cultivated in several countries worldwide, including the Mediterranean region, North Africa, and India."  

**Reviewer Comment 3:** "The USE protocol was selected due to the range of advantages USE presents, including high extraction efficiency and the use of the least possible sample amount, solvent volume, and extraction time." The phrase "the range of advantages USE presents" could be simplified to "the numerous advantages of USE."  

 Response: We agree with the reviewer’s suggestion to simplify this sentence. The revised sentence now reads: "The USE protocol was selected due to the numerous advantages of USE."  

**Reviewer Comment 4:** "The method avoids the need for an extra step of seed crushing and sieving via the use of an ultrasonic dismembrator with small amounts of sample (10 mg) and low solvent volumes." Consider breaking this into two sentences for clarity, as the crushing and sieving highlight the simplicity of the method, while the use of small sample amounts and solvent volumes emphasizes cost-efficiency and eco-friendliness.  

**Response:** We appreciate this suggestion to enhance clarity. The sentence has been split into two, as follows: "The method avoids the need for additional steps, such as seed crushing and sieving, by using an ultrasonic dismembrator. Furthermore, the method employs small sample amounts (10 mg) and low solvent volumes, ensuring cost-efficiency and eco-friendliness."

# Reviewer report: Reviewer response for version 1

**DOI:** 10.5256/f1000research.173145.r340140

**Published:** 2024-11-22

**Authors:** Abdulrahman Koshak

**Affiliations:** 1 King Abdulaziz University, Jeddah, Saudi Arabia

The article presents a novel analytical technique for quantifying the marker compound TG in fenugreek using UPLC-MS/MS. However, it would benefit from a more comprehensive discussion of the disadvantages associated with traditional methods in comparison to the developed approach. For example, elaborating on the advantages of UPLC-MS/MS, the types of solvents commonly used in traditional methods, their potential drawbacks, and how the new method overcomes these challenges would help to better clarify the research gap.

**Title**: Include the keywords "green" and "UPLC-MS/MS" to emphasize the innovative and advantageous aspects of the study.

**Abstract**:

- Clearly articulate the research gap and the drawbacks of traditional methods for quality analysis of fenugreek.
- Avoid mentioning (TG) in brackets, as it has already been identified earlier in the text.
- In the results section, include the units for extract yield and TG concentration for clarity.

**Formatting**:

- Ensure the correct format (UPLC-MS/MS) is consistently used throughout the article.
- Avoid using the symbol "±" for reporting Standard Deviation; opt for a more descriptive format instead.

**Results**:

- Provide the percentages of Trigonelline found in all samples to facilitate comparisons with values reported in the literature.

**Discussion**:

- The first paragraph contains repetitive information regarding the introduction and methodology. Revise this section to focus directly on the findings and the specific research gap addressed by the study.
- Compare your findings for the amount of TG with those in the literature, expressed in percentages, to provide a clearer context and strengthen the relevance of your results.

Is the work clearly and accurately presented and does it cite the current literature?

Yes

If applicable, is the statistical analysis and its interpretation appropriate?

Not applicable

Are all the source data underlying the results available to ensure full reproducibility?

Yes

Is the study design appropriate and is the work technically sound?

Yes

Are the conclusions drawn adequately supported by the results?

Yes

Are sufficient details of methods and analysis provided to allow replication by others?

Yes

Reviewer Expertise:

Pharmacognosy and natural products

I confirm that I have read this submission and believe that I have an appropriate level of expertise to confirm that it is of an acceptable scientific standard, however I have significant reservations, as outlined above.

# Response

**Published:** 2024-11-27

**Authors:** Salma Hago

**Affiliations:** Pharmacognosy, University of Gezira, Sudan

Title Reviewer Comment: Include the keywords "green" and "UPLC-MS/MS" to emphasize the innovative and advantageous aspects of the study. Response: While we appreciate this suggestion, we have chosen to retain the current title because the study has already been published. Changing the title could cause confusion and inconsistency in citations and references to the work. However, we have ensured that the terms "green" and "UPLC-MS/MS" are included in the "Keywords" section to emphasize the innovative and advantageous aspects of the study.

 Abstract Reviewer Comment 1: Clearly articulate the research gap and the drawbacks of traditional methods for quality analysis of fenugreek.

 Response: The abstract has been revised to better articulate the research gap and the limitations of traditional methods, including their inefficiency, reliance on high solvent volumes, and lack of sensitivity.

 Reviewer Comment 2: Avoid mentioning (TG) in brackets, as it has already been identified earlier in the text.

 Response: The brackets around "(TG)" have been removed.

 Reviewer Comment 3: Include the units for extract yield and TG concentration in the results section for clarity.

 Response: Units for extract yield (mg/100mg) and TG concentration (ppb) have been added to the results section of the abstract.

 Formatting Reviewer Comment 4: Ensure the correct format (UPLC-MS/MS) is consistently used throughout the article.

 Response: The manuscript has been reviewed, and we have ensured that "UPLC-MS/MS" is consistently formatted throughout.

 Reviewer Comment 5: Avoid using the symbol "±" for reporting Standard Deviation; opt for a more descriptive format instead.

 Response: The symbol "±" has been replaced with descriptive phrasing (standard deviation, SD).

 Results Reviewer Comment 1: Provide the percentages of Trigonelline found in all samples to facilitate comparisons with values reported in the literature.

 Response: Percentages of Trigonelline in all samples have been added to Table 4. However, direct comparisons with literature values are not feasible because this study focuses on developing a green-sensitive method for trace analysis rather than optimizing an extraction method for high yields. Our approach utilized intact, uncrushed fenugreek seeds, whereas previous studies employed powdered seeds, which likely resulted in significantly lower TG %. Despite this difference, our method provides a rapid and reliable method to detect trace amounts of Trigonelline in fenugreek samples.

 Discussion Reviewer Comment 1: The first paragraph contains repetitive information regarding the introduction and methodology. Revise this section to focus directly on the findings and the specific research gap addressed by the study.

 Response: The first paragraph of the discussion has been revised to eliminate repetitive content. It now focuses on the study's findings and how they address the research gap.  

 Reviewer Comment 2: Compare your findings for the amount of TG with those in the literature, expressed in percentages, to provide a clearer context and strengthen the relevance of your results.

 Response: The findings for TG content, expressed in percentages, have been compared with values from the literature to provide a clearer context and emphasize the significance of our results.
